# *Elephantopus scaber* L. Polysaccharides Alleviate Heat Stress-Induced Systemic Inflammation in Mice via Modulation of Characteristic Gut Microbiota and Metabolites

**DOI:** 10.3390/nu16020262

**Published:** 2024-01-16

**Authors:** Chen Wang, Dongfang Sun, Qi Deng, Lijun Sun, Lianhua Hu, Zhijia Fang, Jian Zhao, Ravi Gooneratne

**Affiliations:** 1 Guangdong Provincial Key Laboratory of Aquatic Product Processing and Safety, College of Food Science and Technology, Guangdong Ocean University, Zhanjiang 524088, China; 18642326533@163.com (C.W.); dfsun@stu.njau.edu.cn (D.S.); suncamt@126.com (L.S.); lianhuashipin@126.com (L.H.); fzj4437549@163.com (Z.F.); 2 College of Food Science and Technology, Nanjing Agricultural University, Nanjing 210095, China; 3 School of Chemical Engineering, The University of New South Wales, Sydney, NSW 2052, Australia; jian.zhao@unsw.edu.au; 4 Department of Wine, Food and Molecular Biosciences, Faculty of Agriculture and Life Sciences, Lincoln University, P.O. Box 85084, Lincoln 7647, New Zealand; ravi.gooneratne@lincoln.ac.nz

**Keywords:** *Elephantopus scaber* L. polysaccharides, heat stress, systemic inflammation, gut microbiota, short chain fatty acids, neurotransmitters

## Abstract

*Elephantopus scaber* L. (ESL) is a Chinese herb that is used both as a food and medicine, often being added to soups in summer in south China to relieve heat stress (HS), but its exact mechanism of action is unknown. In this study, heat-stressed mice were gavaged with ESL polysaccharides (ESLP) at 0, 150, 300, and 450 mg/kg/d^−1^ (*n* = 5) for seven days. The gut microbiota composition, short-chain fatty acids (SCFAs), seven neurotransmitters in faeces, expression of intestinal epithelial tight junction (TJ) proteins (Claudin-1, Occludin), and serum inflammatory cytokines were measured. The low dose of ESLP (ESLL) improved the adverse physiological conditions; significantly reduced the cytokines (TNF-α, IL-1β, IL-6) and lipopolysaccharide (LPS) levels (*p* < 0.05); upregulated the expression of Claudin-1; restored the gut microbiota composition including *Achromobacter* and *Oscillospira*, which were at similar levels to those in the normal control group; significantly increased beneficial SCFAs like butyric acid and 5-HT levels in the faeces of heat-stressed mice; and significantly decreased the valeric acid and glutamic acid level. The level of inflammatory markers significantly correlated with the above-mentioned indicators (*p* < 0.05). Thus, ESLL reduced the HS-induced systemic inflammation by optimizing gut microbiota (*Achromobacter*, *Oscillospira*) abundance, increasing gut beneficial SCFAs like butyric acid and 5-HT levels, and reducing gut valeric and glutamic acid levels.

## 1. Introduction

Excessive exposure to high temperatures causes heat stress (HS) [1]. HS can result in restlessness, poor concentration and anorexia [2]. In severe cases, it can lead to heat stroke, heat exhaustion and intestinal diseases [3]. A series of adverse reactions during HS are closely related to systemic inflammation [1,4]. Therefore, systemic inflammation is often used as a key indicator to evaluate the effectiveness of measures to protect against HS. The current mainstay treatment approaches for HS (such as physical cooling and antibiotics) [5] have various adverse side effects and cannot fundamentally relieve the HS response. Therefore, there is an urgent need to explore safer and more effective intervention methods.

In the subtropical and tropical areas of Southern China and Southeast Asia, *Elephantopus scaber* L. (ESL), a perennial herb [6], is often added to soup and consumed in summer to relieve the physical discomfort caused by high temperatures [7]. Therefore, ESL may have a good intervention effect on systemic inflammation caused by high temperatures. However, there are no scientific studies on the effective components and mechanism of action of ESL in alleviating HS-induced systemic inflammation, limiting its further development and application.

Research has demonstrated that plant polysaccharides can reduce the negative effects of HS on the body. Liu et al. [8] reported that alfalfa polysaccharides improve the growth performance of heat-stressed rabbits. Sohail et al. [9] showed that mannan oligosaccharides improve the relative weight of immune organs in heat-stressed broilers. Similarly, atractylodes polysaccharides are reported to alleviate the splenic inflammatory response induced by HS in broiler chickens [10,11]. These findings suggest that plant polysaccharides may effectively reduce systemic inflammation to protect against HS. Our previous study revealed that ESL extract contains many polysaccharides. Thus, the polysaccharides in ESL may be the effective components underlying the HS-reducing effects of ESL. However, at present, there is a lack of direct evidence, and the mechanism of action requires exploration.

Plant polysaccharides, especially heteropolysaccharides, are generally difficult for the human body to digest and absorb [12]. They can be fermented by the gut microbiota in the large intestine and thus exert their nutritional or pharmacological effects [13]. Plant polysaccharides can improve the health of the body by regulating the composition of the gut microbiota [14]. Research has indicated that fructan (inulin) can improve the diabetic phenotype by enriching *Lactobacillus* and/or *Bifidobacterium* spp. [15]. In another study, fucoidan selectively increased the proportion of *Bacteroides* spp., *Akkermansia muciniphila*, *Blautia* spp. and *Alloprevotella* spp. to ameliorate metabolic syndrome and intestinal malnutrition in mice [16]. However, it is unclear whether *Elephantopus scaber* L. polysaccharides (ESLP) can protect against HS-induced systemic inflammation by regulating the abundance of the particular target gut microbes.

Recent research has demonstrated that polysaccharides can be degraded into short-chain fatty acids (SCFAs) by the intestinal microbiota and that SCFAs play a crucial role in maintaining the health of the body [17]. One study found that *Zizyphus Jujuba cv. Muzao* polysaccharides can significantly increase butyrate and acetate, reducing the risk of colitis-related colon cancer [18]. In another study, *Cyclocarya paliurus* polysaccharides alleviated type 2 diabetes mellitus (T2DM) by increasing the levels of SCFAs (acetic acid, propionic acid, butyric acid, isobutyric acid, valeric acid and isovaleric acid) [19]. It is clear that different polysaccharides can increase the total amount of SCFAs; however, the levels of the increases in specific SCFAs are different, and different SCFAs play different roles. Thus, the effects of ESLP on SCFA production require further clarification. 

In addition, it appears that neurotransmitters produced by the gut microbiota also play a role in inflammation. Feng et al. [20] reported that *Atractylodes macrocephala* Koidz. polysaccharides alleviate dextran sulphate sodium salt-induced ulcerative colitis inflammation by changing the gut microbiota and reversing the reduction in tryptophan. Polysaccharides from the leaves of *Ginkgo biloba* have been found to upregulate the abundance of *Lactobacillus* species and increase the levels of 5-HT in the gut, thus producing anti-inflammatory and antidepressant effects [21]. This suggests that neurotransmitters produced by the intestinal flora are very important in the development of inflammation. However, it is not clear if ESLP can reduce HS-induced systemic inflammation by controlling the neurotransmitters produced by the gut microbiota.

In this study, ESLP was orally administered to heat-stressed mice to investigate the (a) relationships between exposure to HS, systemic inflammation, the gut microbiome, and related SCFAs and neurotransmitters, and (b) the potential mechanism of action underlying the effect of ESLP on systemic inflammation caused by HS.

## 2. Materials and Methods

### 2.1. Extraction of Crude Polysaccharides

Polysaccharides were isolated from *Elephantopus scaber* L. (Product No. PB20200505, a dry powder) purchased from Shaanxi Pioneer Biotech Co., Ltd., Tongchuan, China, according to Zhou’s method with some modifications [22]. Briefly, the crude polysaccharide was extracted via water extraction and ethanol precipitation. The crude polysaccharide solution underwent treatment with a 5% trichloroacetic acid solution to eliminate any remaining protein residues. The supernatant was followed by dialysis using a dialysis bag, then concentrated using a rotary evaporator. After that, lyophilization was used on the concentrated solution, which made *Elephantopus scaber* L. polysaccharides (ESLP). 

### 2.2. Composition Analysis

Total carbohydrates were quantified via the phenol-sulfuric acid method, using glucose as the standard [23]. The reducing sugar content was evaluated via the dinitro salicylic acid (DNS) method [24]. The meta-hydroxy diphenyl technique was used to determine the concentration of uronic acid [25].

### 2.3. Animal Ethics Statement 

The Animal Ethics Committee of Guangdong Ocean University approved all animal tests and methods conducted in this study (file number: GDOU-LAE-2020-015, 18 October 2020). All procedures and experiments were carried out at the laboratory animal centre at Guangdong Ocean University (License No. SYXK 2019-0204) in strict accordance with the university’s laws on animal experimentation. 

### 2.4. Animals and Study Design

The experimental conditions for feeding mice were the same as previously described [26], with some modifications. In total, 25 6-week-old male C57BL/6J mice of average weight (18 ± 2 g) were purchased from Beijing Huafukang Biotechnology Co., Ltd., (Beijing, China). All mice had unlimited access to distilled water and feed pellets sterilised with Co60. High-pressure steam was used to sanitise the materials used for the water bottles, cage, and pads. Three times every week, the water bottles and pad fillings were changed. After a week of acclimatisation, all mice were randomly divided into five groups [Normal Control (NC), HS model (HSS), HS + Low-dose ESLP gavage (150 mg/kg/d^−1^; ESLL), HS + Medium dose ESLP gavage (300 mg/kg/d^−1^; ESLM), HS + High-dose ESLP gavage (450 mg/kg/d^−1^; ESLH)] and housed in different cages (*n* = 5). The NC and HSS group mice were given a normal saline gavage. The NC group was kept in a normal rearing environment (temperature: 22 ± 3 °C, humidity: 60 ± 10%) without HS treatment. The rest of the groups were subjected to HS in an artificial environment simulation cabin to maintain a core temperature of 38 ± 0.5 °C and a humidity of 90–95% [27,28]. The experimental period was 7 days, and the mice were subjected to HS once a day for 4 h. The three ESLP group mice received normal diet plus the ESLP after each HS exposure. 

### 2.5. Temperature-Humidity Index (THI) Measurements

The experimental conditions for constructing the HS model mice were the same as previously described [29], with some modifications. The experimental room ambient temperature (Td, °C) and relative humidity (RH, %) were measured three times each day at 07:00, 13:00, and 20:00. The THI was calculated using the formula below: THI = (1.8 × Td + 32) – (0.55 – 0.55 × RH × 0.01) × (1.8 × Td – 26).

### 2.6. Physiological Variables and Sample Collection

During the experiment, the physiological parameters (body weight, feed intake, body surface temperature, physiological behaviours) of the mice were recorded daily. At the end of the experiment, before dissecting, the mice were massaged on the belly to collect faecal particles, which were placed in sterile centrifuge tubes in an ice bath. The samples were stored at −80 °C. The mice were dissected immediately after 1 mL of eyeball blood was taken. The blood was centrifuged at 1200× *g* and 4 °C for 5 min. The serum samples were separated from the supernatant and kept at −80 °C until required. The organs were collected and weighed. The ileum was immediately collected, and some were washed with precooled phosphate-buffered saline (PBS), frozen in liquid nitrogen, and then stored at −80 °C; others were immediately stored in 10% formalin until analysis.

### 2.7. Measurement of Inflammatory Markers and Lipopolysaccharide (LPS)

A few modifications were made to previous work [26] regarding the measurement of serum inflammatory cytokines and LPS levels. The following mouse ELISA kits, TNF-α (Cat no. M190408-102a), IL-10 (M190408-005a), IL-1β (M190408-001a) and IL-6 (M190408-004a), were purchased from Neobioscience Technology Co., Ltd., Shenzhen, China. The microplate quantitative chromogenic matrix Limulus kit (Cat no. 18030067) was purchased from Xiamen Limulus Reagent Biotechnology Co., Ltd., Xiamen, Fujian Province, China. They were used according to the manufacturer’s instructions.

### 2.8. Haematoxylin and Eosin (H&E) Staining of Intestinal Tissue

Tissues were processed and stained as reported in [26]. Then, 5 μm sections (*n* = 3/animal) were light-microscopically examined for histological damage. The numbers of goblet cells, the villi length and the crypt depth were measured. A total of 5 areas were selected for each sample and an average for each sample and each group was calculated.

### 2.9. Western Blot (WB) Analysis

The experimental conditions for WB were the same as previously described [30], with some modifications. The separated proteins were transferred onto PVDF membranes (Cat no. G6015-0.45, Servicebio, Wuhan, China) using protein extracts (50 μg) from each sample in 10% SDS-PAGE gels. After washing with Tris-Buffered Saline with Tween 20 (TBS-T), the protein bands were detected by chemiluminescence using ECL Western Blot Substrate (Cat no. G2019, Servicebio). β-actin was used as the internal standard. The signals were recorded with a chemiluminescence imager (Cat no. 6300, CLINX, Shanghai, China) and analysed using Alpha Innotech (AlphaEaseFC 4.1.0) and Adobe (Adobe PhotoShop 20.0) software.

### 2.10. Gut Microbiota Analysis

#### 2.10.1. DNA Extraction

Following the manufacturer’s instructions, total gut bacterial genomic DNA samples were obtained using the Metagenomic DNA isolation kit GHFDE100 (Zhejiang Hangzhou Equipment Preparation 20190952) from GUHE Laboratories in Hangzhou, China.

#### 2.10.2. 16S rRNA Amplicon Pyrosequencing and Sequence Analysis

We used 515F (5′-GTGCCAGCMGCCGCGGTAA-3′) as the forward primer and 806R 5′-GGACTACHVGGGTWTCTAAT-3′ as the reverse primer. Details are in a previous publication [26].

#### 2.10.3. Bioinformatics and Statistical Analysis

The experimental bioinformatics and statistical analysis were as described previously [26]. Using the default parameters, linear discriminant analysis effect size (LEfSe) was performed to identify differentially abundant species across groups.

### 2.11. SCFA Analysis

SCFAs in mouse faeces were measured as described previously using GC-MS [26]. 

### 2.12. Neurotransmitter Analysis 

Seven neurotransmitter standards were used. Acetylcholine and Adrenaline were purchased from Sigma, St. Louis, MO, USA. The 5-Hydroxytryptamine (5-HT), Dopamine, γ-aminobutyric acid (GABA), Noradrenaline and Glutamic acid used were from Beijing Suolaibao Technology Co., Ltd. (Beijing, China). The standards were weighed, dissolved in methanol containing 0.2% formic acid and dissolved in acetonitrile/water (2:8) to nine standard concentration gradient mixtures. Faecal samples were thawed on ice prior to extracting polar metabolites for LC-MS analysis. Then, 50 mg of each faecal sample was added into cold water containing 0.2% formic acid. The homogenate was sonicated in an ice bath for 15 min. Next, 1 mL of the homogenate was added to 1 mL of ice-cold 0.2% formic acid in acetonitrile and centrifuged at 4 °C, 12,000× *g* for 3 min. The solution was filtered using an organic microporous membrane of 0.22 µm pore size, and the supernatant was collected and examined using an LC-MS1000 device. The quantitative measurement of faecal neurotransmitters was carried out using a calibration curve. The peak-area integration was assessed based on the retention time.

The neurotransmitters in the faeces of mice were determined via LC-MS. The LC conditions were as follows: Column (TSK-GEL amide-80 column; 4.5 × 150 mm, 5 μm; Tosoh, Tokyo, Japan). The column temperature was 40 °C. Mobile phase A: 100% acetonitrile, Mobile phase B: 0.1% formic acid-water solution (20%:80%). Flow rate: 0.3 mL/min. The MS conditions were as follows: the EI ion source was heated to 450 °C and an ion spray voltage of 4500 V was applied. Data were collected using the ion MRM method. 

### 2.13. Pro-Inflammatory Experiment of Valeric and Glutamic Acid In Vitro

#### 2.13.1. Cytotoxicity Test of Valeric and Glutamic Acid

The experimental conditions for the RAW264.7 cells were the same as previously described [31], with some modifications. The DMEM medium was changed to DMEM medium supplemented with 0, 10, 25, 50, 100, and 200 μg/mL valeric and glutamic acid, respectively.

#### 2.13.2. Pro-Inflammatory Test of Valeric and Glutamic Acid

The experimental conditions for the RAW264.7 cells were the same as previously described [31] with some modifications. The culture medium was changed into DMEM culture medium containing 0, 10, 25, 50, 100, 200 μg/mL valeric and glutamic acid, respectively. After 24 h of cultivation, the concentrations of IL-1β and IL-6 were measured using ELISA kits (Neobioscience Technology Co., Ltd., Shenzhen, China), according to the manufacturer’s instructions.

### 2.14. Statistical Analysis 

The mean and standard error of the mean (SEM) were used to represent the results. SPSS 21.0 (SPSS, Chicago, IL, USA) was used for statistical analysis. One-way analysis of variance (ANOVA) and the LSD test were used to compare the differences between groups, with *p* < 0.05 regarded as statistically significant.

## 3. Results 

### 3.1. Basic Physicochemical Properties of ESLP

The crude polysaccharides of ESL were obtained via hot water extraction and ethanol precipitation. The content (%) of total carbohydrates was 84.69% (including 9.82% reducing sugars), while the uronic acid content was 31.39%.

### 3.2. Effect of ESLP on the Body Weight, Food Intake, Viscera Coefficients, Body Surface Temperature and Physiological Behaviour of Mice

**Figure 1:**
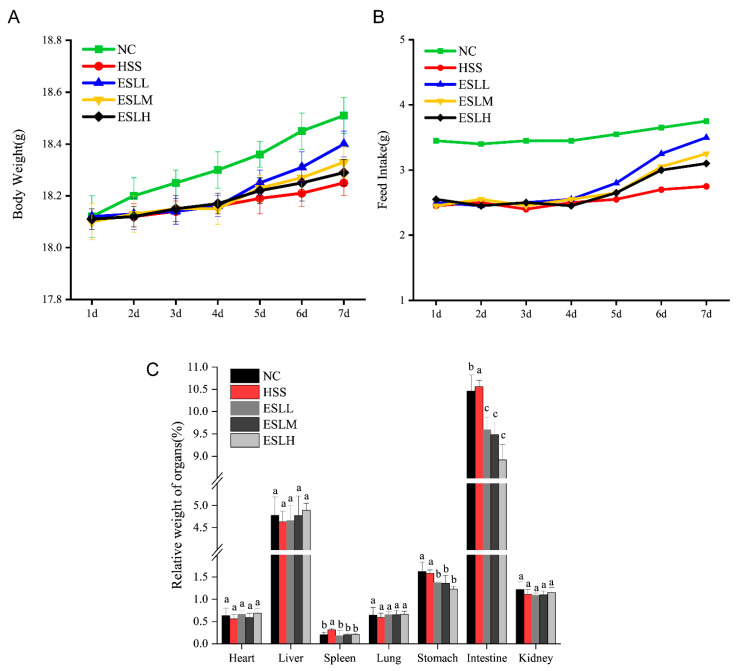
Effects of ESLP on the body weight, food intake and viscera coefficient in heat-stressed mice ((**A**): body weight, (**B**): food intake, (**C**): viscera coefficient). Data are shown as mean ± SE (*n* = 5). Based on the ANOVA statistical analysis, the different letters (a, b, and c) above the bars of each group are statistically different (*p* < 0.05). [Normal control (NC), Heat stress (HS) model (HSS), HS + Low-dose ESLP gavage (ESLL), HS + Medium dose ESLP gavage (ESLM), HS + High-dose ESLP gavage (ESLH)].

The body weight (BW), food intake and viscera coefficients of the mice are shown in Figure 1. Compared with the NC group, the mice in the HSS group exhibited a slower rate of weight gain and decreased food intake. ESLP increased the rate of weight gain and food intake (vs. HSS group). The effect was particularly evident in the ESLL group (Figure 1A,B). Mice in the HSS group had higher spleen and intestine viscera coefficients compared to the NC group. ESLP significantly decreased the viscera indices of the spleen, stomach and intestine of the heat-stressed mice (*p* < 0.05) (Figure 1C). As shown in Table 1, the mice in the HSS group had considerably higher rectal temperatures *(p* < 0.05) than those in the NC group. The ESLL significantly reduced the rectal temperature compared to the HS mice (*p* < 0.05), somewhat similar to the NC group. Although the ESLM and ESLH groups tended to have lower rectal temperatures than the HSS group, these differences were not statistically significant. The HSS group showed increased neck and ear base temperatures compared to the mice in the NC group. Mice in the three ESLP groups exhibited slightly lower neck and ear base temperatures compared to the HS mice. ESLP induced some positive effects with regard to the regulation of the surface body temperature of HS mice, and these effects were particularly visible in the ESLL group.

During the experimental period, the NC group mice were alert, active and healthy, and exhibited dense shiny hair. However, the mice in the HSS group developed the following symptoms from day two onwards: reduced activity, sleepiness, fear, anxiety, irritability, a rapid and irregular breathing rhythm, the visible trembling of legs, coarse hair and severe hair loss. There were significant improvements in these physiological parameters in the groups that received ESLP, indicating that ESLP can ameliorate adverse physiological reactions in HSS group mice. 

### 3.3. Effects of ESLP on Inflammatory Markers and LPS on Heat-Stressed Mice

**Figure 2:**
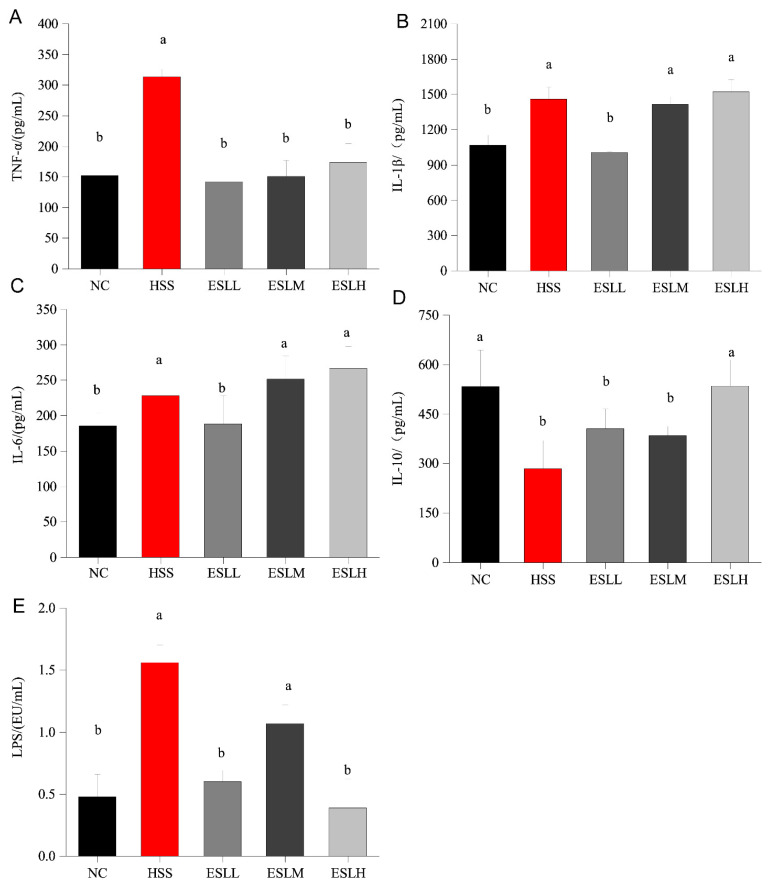
Changes in four serum inflammatory markers and LPS concentrations in mice. (**A**) TNF-α, (**B**) IL-1β, (**C**) IL-6, (**D**) IL-10 (**E**) LPS. Data are shown as mean ± SE (*n* = 5). Based on the ANOVA statistical analysis, the different letters (a, b) above the bars of each group are statistically different (*p* < 0.05). [Normal Control (NC), Heat stress (HS) model (HSS), HS + Low-dose ESLP gavage (ESLL), HS + Medium dose ESLP gavage (ESLM), HS + High-dose ESLP gavage (ESLH)].

The serum concentrations of the pro-inflammatory factors (TNF-α, IL-1β, IL-6) are shown in Figure 2A–C. When compared to the NC group, the HSS group pro-inflammatory factors concentrations were significantly higher (*p* < 0.05); the TNF-α concentration was >two times the NC group. The concentrations of pro-inflammatory factors in the ESLL group were significantly lower than in the HSS group mice (*p* < 0.05), similar to the concentrations in the NC group. The serum concentration of anti-inflammatory factor IL-10 is shown in Figure 2D. The HSS group IL-10 concentration was significantly lower *(p* < 0.05 vs. NC group), whereas in the ESLH group, the IL-10 concentration was significantly higher (*p* < 0.05) than that of the HSS group. The serum LPS concentration in the HSS group was >three times higher than that of the NC group (*p* < 0.01) (Figure 2E). The ESLP treatment significantly reduced the serum LPS concentration in HS mice (*p* < 0.01). This effect was particularly noticeable in the ESLL and ESLH groups. These results indicate that ESLP inhibited inflammation in HS mice, especially in the ESLL. The key indicators that ESLL reduced the systemic inflammation caused by HS were TNF-α, IL-1β, IL-6, and LPS.

### 3.4. Effect of ESLP Treatment on Intestinal Tissue 

**Figure 3:**
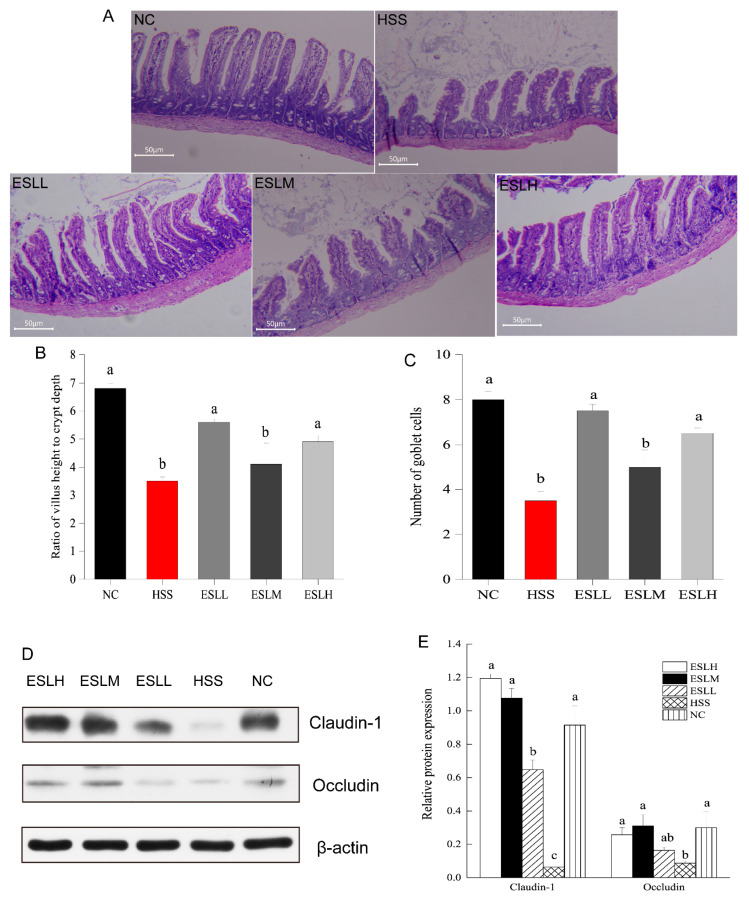
Effect of ESLP on intestinal tissue in HS mice. (**A**): Histological changes observed in the intestinal tissue in different groups; (**B**): Ratio of intestinal villi length to crypt depth; (**C**): mean number of goblet cells in the visual field; (**D**): Western blot of TJ protein; (**E**): Relative expression level of TJ protein. The data are shown as mean ± SE (*n* = 5). The ANOVA statistical analysis shows that the data with distinct letters (a, b, and c) are statistically different in each group (*p* < 0.05). [Normal control (NC), Heat stress (HS) model (HSS), HS + Low-dose ESLP gavage (ESLL), HS + Medium dose ESLP gavage (ESLM), HS + High-dose ESLP gavage (ESLH)].

In Figure 3A, the NC group displayed an ileum mucosa with regularly aligned villi, a typical crypt architecture, a large number of goblet cells, and a condensed configuration of the lamina propria. The ileum mucosa tissue in the HSS group suffered severe damage, including goblet cell depletion, crypt hyperplasia, exposed lamina propria, disrupted intestinal epithelial cell organisation, and the partial loss of villi. The HSS group exhibited a lower ratio of intestinal villi length to crypt depth (*p* < 0.05) (Figure 3B,C). The morphology of the intestinal mucosal in the ESLL and ESLH groups was improved in the HSS group mice. In the ESLL and ESLH groups, the intestinal mucosa was relatively intact, with the crypt morphology, goblet cell numbers and the arrangement of villi and intestinal epithelial cells substantially recovered from HS. In the ESLL and ESLH groups, both the mean number of goblet cells and the ratio of intestinal villi length to crypt depth increased to levels, which were nearly identical to those in the NC group. ESLP treatment affected the intestinal tissue after HS. These effects were particularly evident in the ESLL group.

The impact of ESLP on the tight junction (TJ) proteins occludin and claudin-1 expression in the intestinal tissues of HS mice was assessed via WB. The relative protein expressions of ileum claudin-1 and occludin were significantly lower in the HSS group compared to the NC group (Figure 3D,E; *p* < 0.05). ESLP treatment significantly (*p* < 0.05) increased the expression of TJ proteins to levels comparable to those observed in the NC group. 

### 3.5. Effect of ESLP Treatment on the Structure and Function of the Gut Microbiota in Heat-Stressed Mice

#### 3.5.1. ESLP Maintained the Diversity of the Gut Microbiota in Heat-Stressed Mice

**Figure 4:**
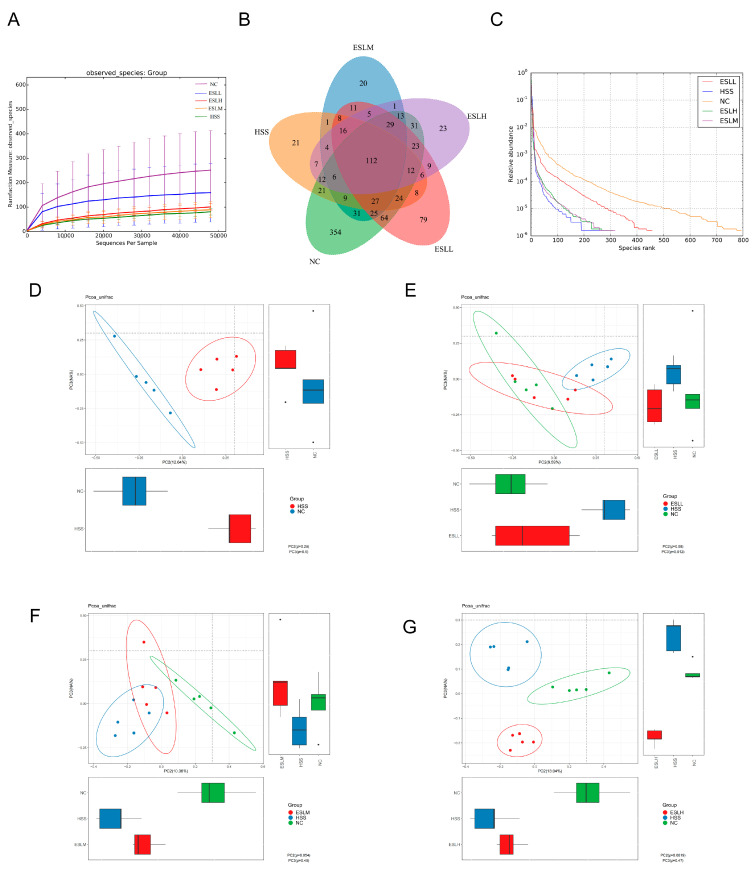
Effects of HS modelling and ESLP treatment on the diversity of gut microbiota in mice. (**A**) Rarefaction Curve; (**B**) Venn diagram based on OTU; (**C**) Rank Abundance curve; (**D**–**G**) PCoA analysis of the gut microbiota of mice. Data are expressed as the mean ± SE (*n* = 5).

ESLL significantly counteracted the HS-induced decline in the species richness and α-diversity of the gut microbiota (Table 2). Unweighted UniFrac distances were used to estimate the beta diversity of the gut flora in the different mouse groups, and PCoA was used to visualise the results (Figure 4D–G). The NC group’s and the HSS group’s confidence ellipses differed significantly from one another (Figure 4D). After the ESLL intervention, the confidence ellipse of this group overlapped with that of the NC group (Figure 4E). However, as the ESLP dose increased, the confidence ellipse gradually deviated from that of the NC group until there was complete separation (Figure 4F,G). The intestinal flora abundance and composition of the ESLL and NC groups were similar. 

#### 3.5.2. ESLP Maintained the Composition of the Gut Microbiota in Heat-Stressed Mice

**Figure 5:**
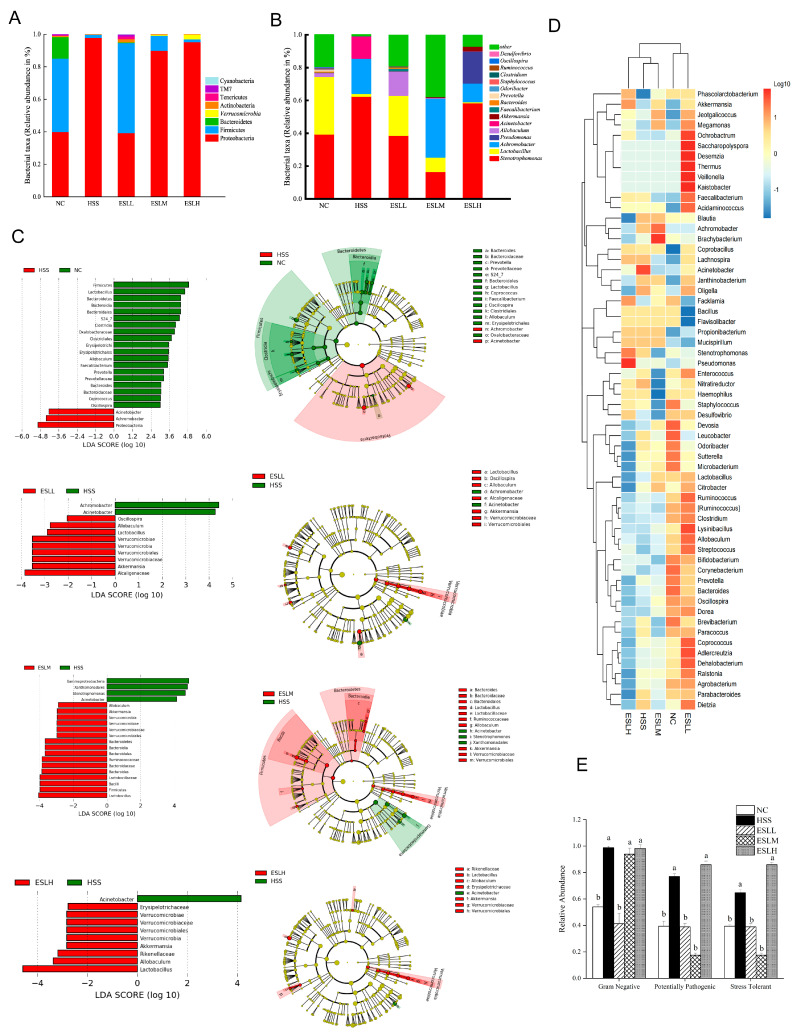
Modelling of the experimental data of the mice gut microbiota. Gut microbiota changes at the phylum (**A**) and genus (**B**) levels. The numerous classification units across groups based on the classification hierarchy tree based on the LEfSe and LDA histograms for taxa showed a significant difference between groups (*p* < 0.05) (**C**). Horizontal clustering heat map of gut microbiota (**D**). Comparison of the relative abundance of gram-negative, potential pathogenic, and stress-tolerant bacteria in each group (**E**). Data are shown as mean ± SE (*n* = 5). Based on the ANOVA statistical analysis, the different letters (a, b) above the bars of each group are statistically different (*p* < 0.05). [Normal control (NC), Heat stress (HS) model (HSS), HS + Low-dose ESLP gavage (ESLL), HS + Medium dose ESLP gavage (ESLM), HS + High-dose ESLP gavage (ESLH)].

The intestinal microflora was examined at two different classification levels. At the phylum level, Proteobacteria, Firmicutes, and Bacteroidetes in the HSS group differed significantly from the NC group (*p* < 0.05). Mice given ESLL showed a significant increase in the abundance of Firmicutes and a similar significant inhibitory effect on the relative abundance of Proteobacteria (*p* < 0.05 vs. HSS group) (Figure 5A). Proteobacteria and firmicutes made up >90% of the total flora. Firmicutes and Bacteroidetes were significantly less prevalent in the HSS group than in the NC group, but proteobacteria were 145% more abundant (*p* < 0.05). In contrast, mice given ESLP showed a significant increase in the abundance of Firmicutes and an equally significant inhibitory effect on the relative abundance of Proteobacteria (*p* < 0.05 vs. HSS group), with the abundances being comparable to those seen in the NC group. The recovery effect on Firmicutes and Proteobacteria did, however, eventually decline when the ESLP dose was increased. The relative abundances of Firmicutes and Proteobacteria in the ESLH group were comparable to those in the HSS group.

*Stenotrophomonas*, *Lactobacillus* and *Achromobacter* were the dominant bacteria of the gut microbiota of each group at the genus level (Figure 5B). Compared with the NC group, the abundances of *Lactobacillus*, *Allobaculum*, *Akkermansia*, *Bacteroides*, *Ruminococcus*, *Faecalibacterium*, *Clostridium*, *Oscillospira*, *Staphylococcus*, *Odoribacter* and *Prevotella* were significantly decreased (*p* < 0.05), while the abundances of *Stenotrophomonas, Achromobacter* and *Acinetobacter* were significantly increased (*p* < 0.05) in the HSS group. This imbalance was restored by the ESLP intervention, where a clear dose–response relationship was observed for *Lactobacillus*, *Akkermansia* and *Allobaculum*, and *Achromobacter*, *Oscillospira*. The abundance of *Clostridium* was restored only by the ESLL intervention. The ESLL group exhibited a similar ratio regarding the relative abundance of gut microbiota to the NC group. 

In order to find species with statistically significant differences between the groups, LEfSe analysis was carried out (Figure 5C). At the genus level, *Achromobacter*, *Acinetobacter*, Lactobacillus, Akkermansia, Allobaculum, Verrucomjcrobia and Oscillospira in the ESLL treatment groups were quite distinct from the HSS group. In the HSS group compared to the NC group, the abundances of gram-negative, potentially pathogenic, and stress-tolerant bacteria were considerably higher (*p* < 0.05) (Figure 5E). When the ESLL group was compared with the HSS group, the abundances of gram-negative, potentially pathogenic, and stress-tolerant bacteria were reduced by 58%, 49% and 40%, respectively, and were comparable to those seen in the NC group. Of note, ESLM did not affect gram-negative bacteria. 

These findings show that the HS-induced gut flora imbalance in mice could be controlled by the ESLP. The effect of ESLL was the most significant (*p* < 0.01), but at higher doses, the effect was lower. The key gut bacteria that ESLL regulated during HS were *Achromobacter*, *Acinetobacter*, *Lactobacillus*, *Akkermansia*, *Allobaculum* and *Oscillospira*.

### 3.6. Effects of ESLP on SCFAs in Heat-Stressed Mice

**Figure 6:**
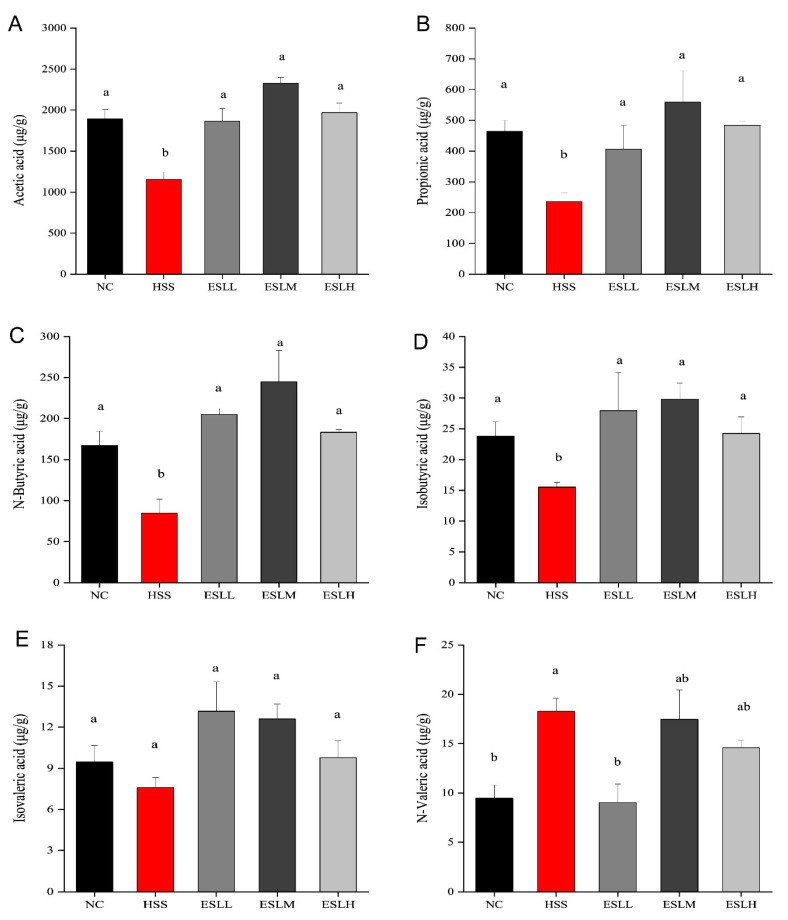
Mice faecal SCFA concentrations. ((**A**): Acetic acid, (**B**): Propionic acid, (**C**): N-Butyric acid, (**D**): Isobutyric acid, (**E**): Isovaleric acid, (**F**): N-Valeric acid). The data are shown as mean ± SE (*n* = 5). The ANOVA statistical analysis shows that the data with different letters a, b shown on top of each column are statistically different from each other (*p* < 0.05). [Normal control (NC), Heat stress (HS) model (HSS), HS + Low-dose ESLP gavage (ESLL), HS + Medium dose ESLP gavage (ESLM), HS + High-dose ESLP gavage (ESLH)].

The concentrations of acetic, propionic, n-butyric, and isobutyric acids were significantly lower (*p* < 0.05) in the HSS group compared to the NC group (Figure 6). In contrast, the n-valeric acid concentration was significantly higher (*p* < 0.05). A lower isovaleric acid concentration was also observed (*p* > 0.05). The acetic, propionic, butyric, and isobutyric acid concentrations in the ESLL, ESLM, and ESLH groups were significantly higher than those in the HSS group (*p* < 0.05), except for isovaleric acid (*p* > 0.05). When compared with the HSS group, the n-valeric acid concentration in the ESLL group was significantly lower (*p* < 0.05), similar to the NC group. As ESLL had a superior inflammation-lowering effect than that shown in the ESLM and ESLH groups, n-valeric acid could be considered an indicator of inflammation caused by HS in ESLL.

### 3.7. Effects of ESLP on Neurotransmitters in Heat-Stressed Mice

**Figure 7:**
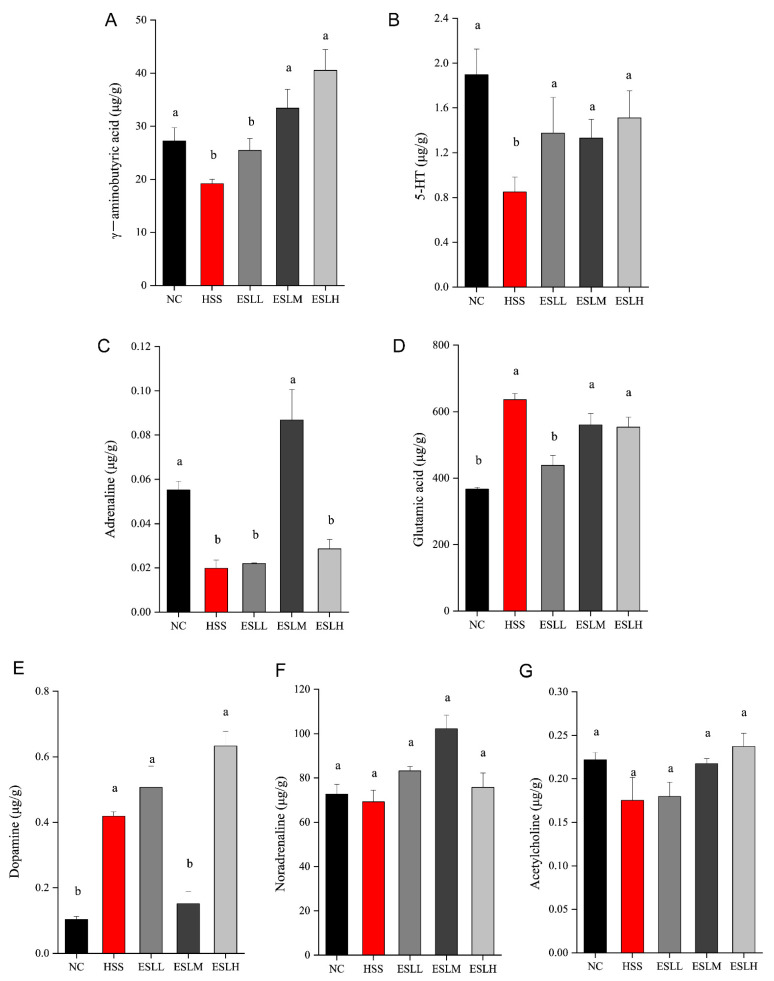
Faecal neurotransmitter concentrations in mice. ((**A**): γ-aminobutyric acid, (**B**): 5-HT, (**C**): Adrenaline, (**D**): Glutamic acid, (**E**): Dopamine, (**F**): Noradrenaline, (**G**): Acetylcholine). Data are shown as mean ± SE (*n* = 5). Based on the ANOVA statistical analysis, the different letters (a, b) above the bars of each group are statistically different (*p* < 0.05). [Normal control (NC), Heat stress (HS) model (HSS), HS + Low-dose ESLP gavage (ESLL), HS + Medium dose ESLP gavage (ESLM), HS + High-dose ESLP gavage (ESLH)].

The GABA, 5-HT, and adrenaline concentrations were significantly lower (*p* < 0.05) in the HSS group compared to the NC group, while the glutamic acid and dopamine concentrations were significantly higher (*p* < 0.05) (Figure 7). Compared with the HSS group, the concentrations of 5-HT were significantly increased (*p* < 0.05) but the glutamic acid concentration was significantly lower (*p* < 0.05) in the ESLL group. These results suggest that ESLL plays a role in regulating the concentration of neurotransmitters in HS mice.

### 3.8. Correlation Analysis of Inflammation Markers, SCFAs, Neurotransmitters and Gut Microbiota

**Figure 8:**
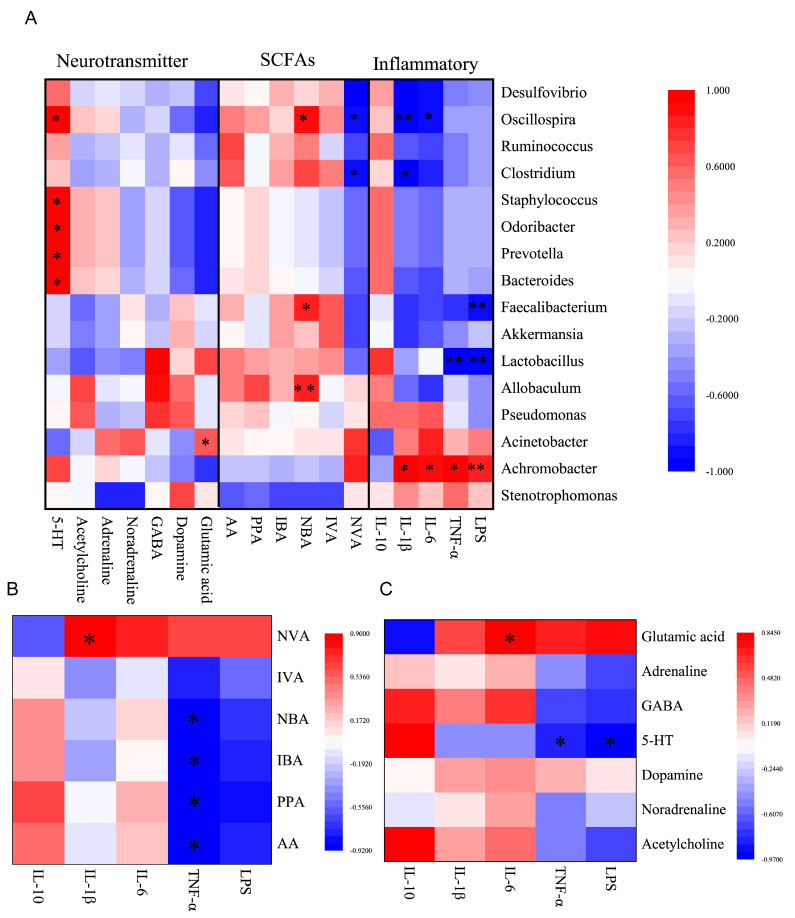
Correlation analysis of inflammatory markers, short-chain fatty acids (SCFAs), neurotransmitters and gut microbiota. (**A**): Relationship between the gut microbiota and inflammation, neurotransmitters, and SCFAs. (**B**): Correlation between SCFAs and inflammatory markers. (**C**): Correlation between neurotransmitters and inflammatory indicators. * *p* < 0.05, ** *p* < 0.01. AA: Acetic acid, PPA: Propionic acid, NBA: N-Butyric acid, IBA: Isobutyric acid, IVA: Isovaleric acid, NVA: N-Valeric acid. [Normal control (NC), Heat stress (HS) model (HSS), HS + Low-dose ESLP gavage (ESLL), HS + Medium dose ESLP gavage (ESLM), HS + High-dose ESLP gavage (ESLH)].

To determine possible intrinsic associations between the serum inflammatory marker levels, faecal SCFAs levels, faecal neurotransmitters levels, and the abundance of the faecal gut microbiota, a correlation analysis between these indicators was conducted using Pearson’s correlation analysis (Figure 8). The correlation between the key indicators showing that ESLL reduces systemic inflammation caused by HS was particularly analysed. *Oscillospira* positively correlated with butyric acid and negatively correlated with IL-6 and IL-1β; *Achromobacter* positively correlated with LPS, TNF-α, IL-6 and IL-1β; Acetic, propionic, butyric and isobutyric acid negatively correlated with TNF-α; Valeric acid positively correlated with IL-1β; 5-HT negatively correlated with LPS and TNF-α; and Glutamic acid positively correlated with IL-6.

### 3.9. Pro-Inflammatory Activity of Valeric and Glutamic Acid In Vitro

**Figure 9:**
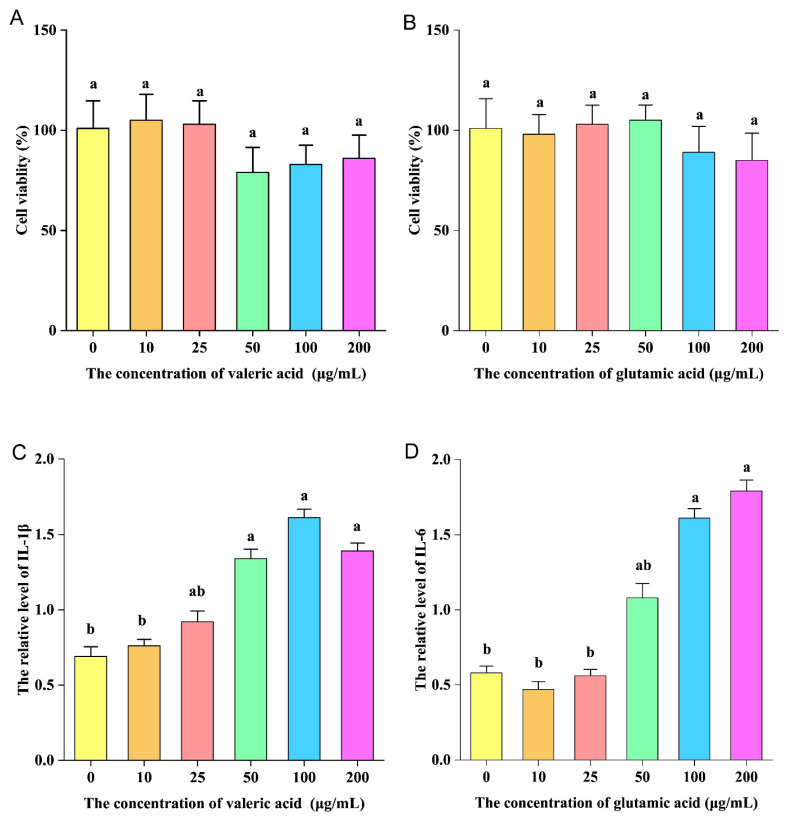
(**A**,**B**): The effects of different concentrations of valeric and glutamic acid on the survival rate of RAW264.7 macrophages. (**C**) The effect of different concentrations of valeric acid on IL-1β production in RAW264.7 macrophages. (**D**) The effect of different concentrations of glutamic acid on IL-6 production in RAW264.7 macrophages. The ANOVA statistical analysis shows that the data with different letters a, b shown on top of each column are statistically different from each other (*p* < 0.05).

Valeric and glutamic acid were selected for pro-inflammatory experiments in vitro. In Figure 9A,B, it can be seen that valeric and glutamic acid had no obvious cytotoxicity to RAW264.7 cells at a concentration of 10–200 μg/mL. Valeric acid significantly increased the level of IL-1β produced by RAW264.7 cells when the concentration was 50 μg/mL (Figure 9C). Glutamic acid significantly increased the level of IL-6 produced by RAW264.7 cells when the concentration was 100 μg/mL (Figure 9D). This indicates that valeric and glutamic acid have pro-inflammatory activity in vitro.

## 4. Discussion

ESL is commonly added to the diet to regulate physical health [7], and has been widely studied in the scientific community [32,33]. It is known that ESL has many bioactive components, including polyphenols, flavonoids and sesquiterpene lactones [6,34]; however, the function of the polysaccharides in ESL has not yet been elucidated. In this study, the effect of ESLP on HS was investigated in heat-stressed model mice. The apparent indicators, including systemic inflammation, the gut microbiota, and related metabolites, were investigated, with the findings offering evidence of the beneficial effects of ESLP in the alleviation of systemic inflammation induced by HS and the possible targets underlying its effects.

The results of this study demonstrated that both low, medium and high doses of ESLP improved the HS-induced inflammatory response to different degrees, and that there were no significant toxic side effects on the body. A comprehensive analysis of body weight, feed intake, the serum inflammation level, intestinal tissue integrity, the gut microbiota and related metabolites, and other indicators revealed that ESLL was significantly more effective than ESLM and ESLH in improving HS. This suggests that the effect of ESLP on HS does not increase with increases in the dosage; an appropriate dosage can have a good anti-inflammatory effect. This may be because different doses of ESLP may have different effects on the gut microbiota and its metabolites; this, in turn, affects the body’s immune system and leads to different levels of inflammation [35]. Other plant polysaccharides should have similar physiological effects [36]. The current findings offer a reference for future studies to investigate suitable doses of plant polysaccharide-based dietary supplements.

To date, various polysaccharides have been reported to alleviate HS. For example, alfalfa polysaccharides improve the growth performance of heat-stressed rabbits [8], atractylodes polysaccharides alleviate the splenic inflammatory response induced by HS in broiler chickens [10,11], and mannan oligosaccharides have a protective effect on HS-induced liver injury in broilers [37]. However, the above studies only focused on the local damage induced by HS and did not elucidate the mechanism by which polysaccharides alleviate HS responses in terms of key targets, such as systemic inflammation, the gut microbiota and related metabolites. Therefore, there is great potential to develop ESLP into products that resist HS responses.

Studies have shown that there are significant differences in the regulatory effects of different plant polysaccharides on the gut microbiota [15,38]. However, the role of polysaccharides in the regulation of key gut microflora during HS has rarely been reported. This study found that ESLL, like many polysaccharides, up-regulated the abundance of *Lactobacillus*, *Allobaculum* and *Akkermansia* to play a beneficial regulatory role [20]. However, inconsistent with previous findings, ESLL also significantly increased the abundance of *Oscillospira*, which was decreased due to HS, and significantly decreased the abundance of *Achromobacter*, which was increased due to HS. This suggests that *Achromobacter* and *Oscillospira* may be key target gut microbes that play a role in the regulatory effect of ESLL on HS.

Moreover, this study found, for the first time, that HS induced the massive proliferation of *Achromobacter.* However, following the ESLL intervention, the abundance of *Achromobacter* was significantly reduced. It has been reported that *Achromobacter* is an opportunistic pathogen [39], and its proliferation can induce damage to the intestinal barrier [40], leading to the entry of LPS from the gut into the bloodstream, which can trigger systemic inflammation [41]. This is consistent with the results of this study, which showed significant positive correlations between serum LPS, TNF-α, IL-6, and IL-1β levels and the abundance of *Achromobacter*. This suggests that *Achromobacter* is likely to be a key bacterium in HS-induced systemic inflammation. However, the reasons why HS triggers the proliferation of *Achromobacter* are unknown and need to be further investigated. In this study, based on the effects of the low, medium and high-dose ESLP interventions on the changes in the gut microbiota associated with HS, it is speculated that ESLL can reverse the increase in *Achromobacter*, probably because gut microbes such as *Lactobacillus*, *Akkermansia* and *Allobaculum*, which are the nutritional sources of ESLP, have the opportunity to occupy the ecological niche, resulting in a reduction in *Achromobacter*.

It is also possible that metabolites produced by the fermentative metabolism of ESLL induced by the gut flora may alter the environmental conditions in the gut, including the pH, oxygen level or other environmental parameters, to the detriment of *Achromobacter*, thus reducing systemic inflammation [42].

*Oscillospira* is an intestinal bacterium that can ferment complex carbohydrates and produce beneficial SCFAs such as butyric acid [43,44], which play an important role in the health of organisms. Feng et al. reported that pueraria lobata polysaccharides, like ESLL, can also significantly increase *Oscillospira* abundance, thereby attenuating antibiotic-associated diarrhoea (AAD)-induced colonic pathology and the ecological dysbiosis of mice intestinal flora [45]. However, the exact mechanism is unclear. In the current study, the correlation results revealed that the abundance of *Oscillospira* was significantly positively correlated with the butyric acid level and significantly negatively correlated with the serum inflammatory cytokine levels (IL-6 and IL-1β levels). These findings provide insight into the possible mechanism by which ESLP can alleviate HS-induced systemic inflammation. Specifically, *Oscillospira* will proliferate using ESLP as a nutrient source and will metabolise butyric acid, which is involved in the regulation of intestinal barrier integrity [46], thereby reducing the inflammatory response. Due to the relative complexity of the mechanisms by which plant polysaccharides affect the gut microbiota and the multifaceted effects of ESLP on the gut microbiota, the mechanisms of action of ESLP require further study.

SCFAs are important intestinal microbial metabolites that play a crucial role in the regulation of intestinal barrier integrity and immune factors [47]. Increases in propionate and butyrate can regulate immune homeostasis and fight against inflammation [17,19]. Increases in butyrate and acetate can reduce damage to colonic tissue and the risk of colitis [18]. This suggests that increased levels of acetic, propionic and butyric acid play a key role in reducing inflammation. This is consistent with the correlation results of this study: the serum TNF-α level was significantly negatively correlated with the acetic, propionic, butyric and isobutyric acid levels. However, in contrast to existing studies, in the current study, HS significantly increased the faecal valeric acid level and, following the ESLL intervention, this increase in the level of valeric acid was reversed. It is widely believed that valeric acid has positive effects on the body [48]. However, the results of the current study indicated that the level of the serum pro-inflammatory factor IL-1β was significantly positively correlated with the faecal valeric acid level. Further, our in vitro analysis demonstrated that valeric acid had pro-inflammatory effects on RAW264.7 cells. This suggests that valeric acid may sometimes cause inflammation. This effect may be closely linked to the amount of valeric acid present and the activation pathway of certain inflammatory cells. More research is needed to fully understand this mechanism.

Another interesting finding in this study was that ESLL restored gut barrier integrity in heat-stressed mice without maximising TJ protein expression. It is widely accepted that TJ protein expression promotes gut barrier integrity and that the two should be positively correlated. However, one study reported that valeric acid produced the biggest increase in transepithelial electrical resistance (TEER) and reduced paracellular permeability [49]. This activity was not linked to the reinforced expression of TJ-related proteins [49]. This finding also suggests that the ability of ESLL to restore gut barrier integrity in HS model mice is not only related to SCFAs such as butyric acid, which can provide energy to the gut barrier; valeric acid also appears to play an important role in this effect. Therefore, we speculate that valeric acid could be a potential indicator of HS.

Compared with the number of studies that have investigated the effects of polysaccharides on SCFAs, a smaller number of studies have investigated the effects of polysaccharides on other gut microbiota metabolites, such as neurotransmitters. Gut bacteria can produce a variety of neurotransmitters identical to those produced by the host. They can be absorbed by the gut to perform corresponding regulatory functions [50]. In this study, HS significantly reduced the faecal 5-HT concentration and, following the ESLL intervention, this effect was significantly reversed. Moreover, the faecal 5-HT level was significantly negatively correlated with the serum LPS and TNF-α levels. The available studies generally indicate that increased levels of 5-HT will affect the immune system by stimulating T-cell proliferation [51] and activating dendritic cells [52]. However, Koopman and Katsavelis [53] reported that 5-HT contributes to the strengthening of the gut barrier, which is essential for maintaining intestinal homeostasis and reducing inflammation. Thus, the role of 5-HT in inflammation is not entirely clear, as it can be either pro- or anti-inflammatory. Accordingly, the relationship between gut flora-induced changes in 5-HT levels and HS-induced systemic inflammation requires further exploration.

This study also found that the glutamic acid concentration was significantly higher in the HSS group compared to the NC group. It is widely believed that glutamic acid is a metabolite of many desirable gut bacteria, such as *Lactobacillus* and *Bifidobacterium*. Glutamic acid can assist in detoxification by removing toxic metabolic waste products [54], thus aiding in nutrient absorption and the maintenance of a healthy gut lining [55]. However, the results of this study indicated that the faecal glutamic acid level was significantly positively correlated with the serum pro-inflammatory factor IL-6 level. Further, the in vitro analysis demonstrated that glutamic acid had a pro-inflammatory effect on RAW264.7 cells. Therefore, it can be speculated that, during HS, the gut flora metabolise high levels of glutamate which, in turn, stimulates IL-6 production, thereby triggering inflammation. However, another study reported that glutamic acid decreases IL-6 production in vitro [56]. Thus, the relationship between glutamic acid and IL-6 production is complex. Further research is needed to fully understand the mechanisms and effects of glutamic acid on IL-6 production. The findings of this study also indicated that the ESLL intervention significantly reduced the glutamic acid levels. These findings imply that ESLL can alleviate HS-induced systemic inflammation by regulating the glutamic acid concentration. Thus, it can be speculated that ESLL may directly inhibit the abundance of specific gut flora involved in amino acid metabolism or that substances produced by ESLL via the metabolism of intestinal flora may inhibit glutamate synthase or glutamate metabolism for reasons that need to be further explored. Taken together, the current findings suggest that glutamic acid may also be a potential marker of HS systemic inflammation.

In summary, this study investigated the possible mechanisms by which ESLL alleviates HS-induced systemic inflammation. These findings offer a novel perspective and a viable plan for identifying potential indicators of HS inflammation and developing strategies to prevent and manage this condition. These findings also offer a theoretical basis for the ongoing development of ESLP into effective functional products that prevent and control HS, and for the safe and effective utilisation of related plant polysaccharides. This study will be followed up with a comprehensive study of ESLP, including its chemical components, molecular mass and structure, in order to fully understand the relationship between the structure and efficacy of ESLP. The ultimate goal is to develop ESLP and its structurally similar polysaccharides into functional ingredients or foods to promote health.

## 5. Conclusions

This study revealed that ESLL alleviates HS-induced systemic inflammation in mice by reducing serum TNF-α, IL-6 and IL-1β levels according to three main mechanisms: (a) ESLL supplementation regulates the gut microbiota, promotes the proliferation of beneficial bacteria (*Lactobacillus*, *Akkermansia* and *Allobaculum*), and inhibits the proliferation of harmful bacteria (*Achromobacter*), thus reducing the LPS level. (b) ESLL promotes the gut microbiota (*Oscillospira*), which produces beneficial SCFAs like butyric acid, provides sufficient energy for gut epithelial cells, and strengthens the intestinal barrier, making it harder for harmful substances to move into the bloodstream. (c) ESLL regulates gut microbiota metabolites, thus increasing 5-HT levels and decreasing valeric and glutamic acid levels.

## Figures and Tables

**Table 1:** Effect of ESLP on the body surface temperature in heat-stressed mice (Mean ± SE).

Temperature	NC	HSS	ESLL	ESLM	ESLH
ear base/°C	36.07 ± 0.29 ^a^	36.20 ± 0.08 ^a^	36.12 ± 0.25 ^a^	36.02 ± 0.20 ^a^	35.96 ± 0.23 ^a^
neck/°C	36.40 ± 0.11 ^a^	36.46 ± 0.19 ^a^	36.44 ± 0.17 ^a^	36.46 ± 0.11 ^a^	36.42 ± 0.19 ^a^
rectal/°C	35.62 ± 0.10 ^b^	35.94 ± 0.13 ^a^	35.57 ± 0.06 ^b^	35.87 ± 0.05 ^a^	35.70 ± 0.25 ^a^

Data are shown as mean ± SE (*n* = 5). In the same row, data with different small letter (a, b) superscripts are significantly different (*p* < 0.05) based on the ANOVA statistical analysis. [Normal control (NC), Heat stress (HS) model (HSS), HS + Low-dose ESLP gavage (ESLL), HS + Medium dose ESLP gavage (ESLM), HS + High-dose ESLP gavage (ESLH)].

**Table 2:** Effect of ESLP on the gut microbiota α-diversity index of the heat-stressed mice (Mean ± SE).

Groups	Shannon	Simpson	Chao1	ACE	Goods Coverage
NC	1.960 ± 0.911 ^a^	0.662 ± 0.219 ^a^	263.414 ± 86.015 ^a^	265.013 ± 81.011 ^a^	1.000 ± 0.000 ^a^
HSS	1.457 ± 0.411 ^ab^	0.465 ± 0.045 ^a^	172.756 ± 25.611 ^ab^	163.025 ± 30.418 ^b^	1.000 ± 0.000 ^a^
ESLL	1.847 ± 1.220 ^a^	0.647 ± 0.111 ^a^	212.085 ± 100.941 ^ab^	203.030 ± 89.117 ^a^	1.000 ± 0.000 ^a^
ESLM	0.557 ± 0.139 ^b^	0.126 ± 0.037 ^b^	161.470 ± 42.782 ^b^	168.618 ± 37.352 ^b^	1.000 ± 0.000 ^a^
ESLH	1.526 ± 0.323 ^ab^	0.565 ± 0.028 ^a^	202.863 ± 25.556 ^ab^	191.318 ± 24.231 ^ab^	1.000 ± 0.000 ^a^

Data are shown as mean ± SE (*n* = 5). In the same column, data with different small letter (a, b) superscripts are significantly different in different groups (*p* < 0.05) based on the ANOVA statistical analysis. [Normal control (NC), Heat stress (HS) model (HSS), HS + Low-dose ESLP gavage (ESLL), HS + Medium dose ESLP gavage (ESLM), HS + High-dose ESLP gavage (ESLH)].

## Data Availability

The corresponding author will provide the information supporting the findings upon reasonable request. The data are not publicly available due to privacy reasons.
